# Foreign Bodies in the Ear

**Published:** 1878-06

**Authors:** 


Foreign Bodies in the Ear.—There are a few broad rules which ought to be remembered by any one attempting to extract a foreign body from the ear. They are as follows: 1. See the body; make sure that there is a foreign body present. This, as a rule, is easily done where there has been no previous inflammation. 2. Remember that a body which will not swell and has no cutting edge will generally remain without causing any urgent symptoms. 3. Warm water injection is the best of all methods of removing foreign bodies. 4. If it be a vegetable substance, do not inject fluid unless you have time to extract the body either at the one operation or shortly afterwards. 5. Injection failing, which is very exceptional, a surgeon with the necessary appliances ought to be at once consulted, as, should urgent symptoms arise from the irritation in the attempted extraction by incisions, galvano-cautery, boring out the centre of the substance and so causing it to collapse, or even detachment of the posterior attachment of the auricle may be necessary. 6. To attempt to extract a substance without seeing it is highly dangerous.—(*British Med. Journal.*)—*Toledo Medical Journal.*

